# Using Vehicle Interior Noise Classification for Monitoring Urban Rail Transit Infrastructure

**DOI:** 10.3390/s20041112

**Published:** 2020-02-18

**Authors:** Yifeng Wang, Ping Wang, Qihang Wang, Zhengxing Chen, Qing He

**Affiliations:** 1 Key Laboratory of High-Speed Railway Engineering of the Ministry of Education, School of Civil Engineering, Southwest Jiaotong University, Chengdu 610031, China; yfw@my.swjtu.edu.cn (Y.W.); wping@swjtu.edu.cn (P.W.); qihangwang@my.swjtu.edu.cn (Q.W.); chenzhengxing@my.swjtu.edu.cn (Z.C.); 2 Department of Industrial and Systems Engineering, University at Buffalo, The State University of New York, Buffalo, NY 14260, USA; 3 Department of Civil, Structural and Environmental Engineering, University at Buffalo, The State University of New York, Buffalo, NY 14260, USA

**Keywords:** urban rail transit interior noise, smartphone sensing, XGBoost classifier, railway maintenance

## Abstract

This study developed a multi-classification model for vehicle interior noise from the subway system, collected on smartphones. The proposed model has the potential to be used to analyze the causes of abnormal noise using statistical methods and evaluate the effect of rail maintenance work. To this end, first, we developed a multi-source data (audio, acceleration, and angle rate) collection framework via smartphone built-in sensors. Then, considering the Shannon entropy, a 1-second window was selected to segment the time-series signals. This study extracted 45 features from the time- and frequency-domains to establish the classifier. Next, we investigated the effects of balancing the training dataset with the Synthetic Minority Oversampling Technique (SMOTE). By comparing and analyzing the classification results of importance-based and mutual information-based feature selection methods, the study employed a feature set consisting of the top 10 features by importance score. Comparisons with other classifiers indicated that the proposed XGBoost-based classifier runs fast while maintaining good accuracy. Finally, case studies were provided to extend the applications of this classifier to the analysis of abnormal vehicle interior noise events and evaluate the effects of rail grinding.

## 1. Introduction

By the end of 2018, the total operating mileage of urban rail transit (URT) in China exceeded 5700 km, including 4350 km of subway lines, and it is expected to double in the next 3 to 5 years [1]. With the rapid extension of the URT network, the current maintenance mode relies on humans, and it is challenging to ensure the safe and stable operation of trains. Therefore, intelligent URT maintenance work should be promoted for higher efficiency.

As one of the most prevalent kinds of URT, subways are increasingly essential in people’s daily lives. However, abnormal vibration and noise significantly affect passengers’ riding experience. Moreover, these abnormalities provide information about wheel-rail interactions and degradation of the track structures. Generally, train-induced noise can be categorized as external or interior noises [2]. Vehicle interior noise which is pertinent to this study mainly consists of noise from electrical equipment, aerodynamic noise, and wheel-rail noises [3]. Usually, the aerodynamic noise is dominant when the train speed exceeds 250 km/h, and electrical equipment noise dominates for speeds slower than 35 km/h [4]. As the subway trains usually run at 30–80 km/h, the wheel-rail noise is the main component of vehicle interior noise [5]. The wheel-rail interaction significantly influences the wheel-rail noise. Therefore, we assumed that there exists a mapping relationship between vehicle interior noises and wheel-rail interactions. This mapping relationship provides an approach to monitor track conditions through vehicle interior noise. Moreover, it would be convenient to develop a simple onboard interior noise monitoring system that contributes to the safety and reliability of the railway system.

Regarding vehicle interior noise, past studies have mainly focused on the generation mechanism, transmission characteristics, and control strategies [6,7,8,9,10]. Typical study topics, such as noise characteristics analysis [11], sound quality evaluation [12], and noise level prediction [13], can be attributed to the above research fields. However, because the vehicle-track coupling system consists of a large number of components, the interior noise is affected by numerous factors, such as track slab [14], rail roughness, wheel out-of-roundness [9], and car body structure [15]. These factors may interact with each other and influence the characteristics of vehicle interior noise. Therefore, researchers generally choose one or two factors, such as rail fastener stiffness [7] and wheel polygonal wear [9], to perform their analysis at a lower complexity.

Among related studies, the prediction of vehicle interior noise is one of the most prevalent topics because it benefits the design and construction of track-vehicle systems at the early stages. Methods such as the boundary element method (BEM) [16], finite element method (FEM) [17], and statistical energy analysis method (SEAM) [15] are commonly used in this. However, their effectiveness relies significantly on the selected boundary conditions and model parameters. Thus, these numerical models are generally applied for specific problems. Moreover, the results of field tests are also often used for model verification. Despite the effectiveness of the method combining analytical models, numerical simulation, and field tests in the study of vehicle interior noise, the difficulty to obtain model parameters limits its application. Moreover, field tests may also interfere with daily operations. Overall, these studies do not make the best use of data collected during the daily operation and maintenance of the railway system.

In this context, the railway transportation industry is at the forefront of implementing analytics and big data [18]. Machine learning (ML) and artificial intelligence (AI) are two concepts at the leading edge of information technology, both of which contribute to big data technology. In recent years, the implementation of ML in the railway industry has been widely studied, for example in the prediction of passenger flow [19], delay events [20], and railway operation disruptions [10]. Moreover, many cases have been reported for railway infrastructure management and maintenance, including the detection and diagnosis of defects [21,22,23], prediction of failure events [24,25], and forecast of remaining useful life of devices [26]. These studies indicate that ML technologies have a promising prospect in promoting intelligent railway maintenance, thus ensuring the safety of the railway transit system.

As for data on vehicle interior noise, users require automatic methods to segment, label, and store the increasing amount of acoustic data from monitoring systems. The major challenge in this field is the automatic classification of audio [27]. Recent studies on the classification of traffic noise have been conducted, for example, to identify the type of vehicle through roadside noise [28,29] and evaluate passengers’ subjective experience by categorizing the cabin’s interior noise [30]. However, compared with traffic noise, the factors influencing vehicle interior noise of subway trains are considerably more complicated.

For collecting track conditions, the railway industry has employed various dedicated devices, such as track inspection vehicles [31] and visual inspection systems [32]. Although these devices perform well in detecting track conditions, the expensive cost and the interference for regular operation limit their usage in urban rail transit systems. There are also some on-board devices being developed to monitor track conditions using in-service vehicles [33,34,35]. However, the installation of these devices may change the design characteristics of cars and cause potential safety issues. As of now, these novel on-board monitoring devices have not been widely used. As an integrated platform, a smartphone can achieve data collection, storage, and transmission individually. Besides, the smartphone is mature, cost-effective, and easy to use, promoting its application in various fields. Studies using the embedded accelerometers of smartphones to monitor road conditions and evaluating the ride quality have been reported [36,37]. These research works inspired the authors to investigate the feasibility of using smartphones to collect multi-source data about subway vehicles.

According to the above literature review, current studies about vehicle interior noise mainly focus on its generation mechanism and influencing factors through analytical models, numerical simulations, and field tests. To the best of our knowledge, only a few studies have analyzed vehicle interior noise using data-driven methods. Therefore, this study aims to advance data mining of vehicle interior noise for decision making in rail maintenance, such as for rail grinding. In this context, there are two significant challenges. First, despite sensing technologies being well developed now, it is still difficult to establish an onboard data collection framework that is easy to deploy, cost-efficient, and reliable. Moreover, the simultaneous collection of dynamic responses from the car body and interior noise is essential because these two datasets are connected to each other. Second, due to the complexity of vehicle interior noise, the extraction of useful features and correct labeling of noise classes remain challenging.

The goal of this study is to mine useful information from the vast amount of interior noise data using ML methods. To pursue this goal, onboard smartphone data were collected, including dynamic responses and noises. Further, a series of analyses were performed to classify the noises and clarify the influencing factors. The novel contributions of this paper are summarized as follows:

1. A smartphone-based onboard data collection framework for vehicle interior noise and dynamic responses of the car body was established.
2. The theory of Shannon entropy was considered when selecting the optimal window size for segmenting the multi-source time-series signals.
3. A multi-classification model for subway vehicle interior noise was established based on the XGBoost algorithm. The generation of a set of 45 features and performing feature selection based on different methods were also included.
4. Case studies were conducted to extend the application scenario for the analysis of abnormal noise causes and evaluating the effect of rail grinding.

This paper is organized as follows. Section 2 briefly illustrates the research methodology. Section 3 introduces the data utilized in this study and its collection framework. Section 4 describes the modeling approaches, including data segmentation and time windows, and establishes the multi-classification model with the Extreme Gradient Boosting (XGBoost) method. Furthermore, Section 5 presents the analysis results and discussions. Finally, in Section 6, conclusions are drawn according to the relevant analysis.

## 2. Research Methodology

**Figure 1:**
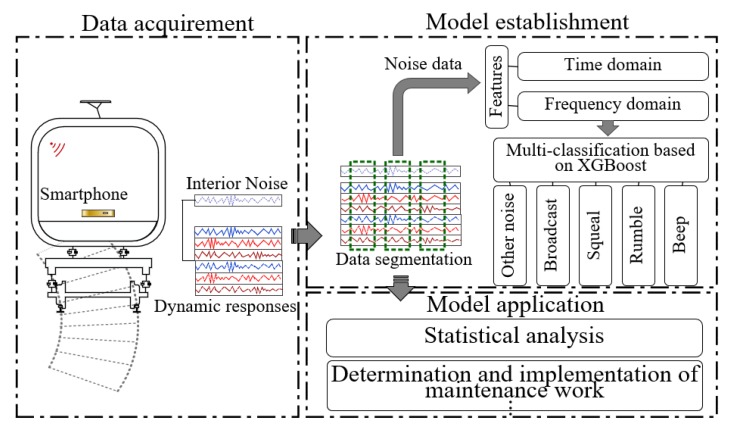
Research methodology of this study.

The research methodology of this study is shown in Figure 1. First, we developed an Android app that leverages built-in sensors of onboard smartphones to collect vehicle interior noise and the corresponding dynamic responses of the car body. Second, time windows were used to segment the multi-source signals and establish the corresponding relationship between the audio and other signals. This method was significantly effective in overcoming the difficulty brought by the different sampling frequencies of a variety of sensors. Third, features were generated and selected from the time- and frequency-domains. Fourth, an automatic classification model for train interior noise was developed using XGBoost, a tree-based method. Finally, the proposed model was validated based on field experiments on the subway line.

## 3. Data Collection and Description

**Figure 2:**
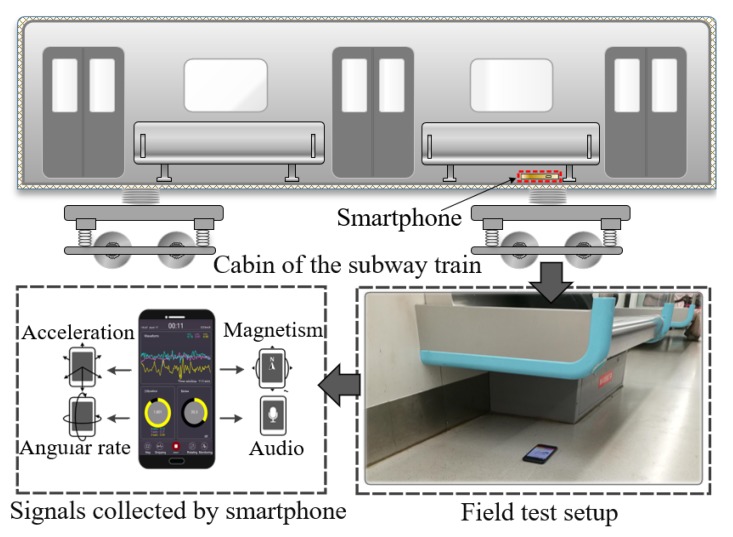
Data collection with the smartphone.

Figure 2 shows the field test setup for data collection using Android smartphones (Huawei Honor FRD-AL00). During the test, the smartphone was placed on the cabin floor, right above the bogie to sense the response from the wheel-rail contact interface. In a parallel study, we verified that the differences between smartphone sensors and high-precision industry accelerators are acceptable, especially in the vertical direction [36]. Thus, the dynamic response signals can be considered a good record of the movement state of the car body. An app was developed to save and transmit the data to our cloud server. In the field test, three sensors were used, namely the microphone, accelerometer, and gyroscope. Moreover, considering the performance of these sensors and the characteristics of the signals, the sampling frequency of the accelerometer and gyroscope were set to 100 Hz, and that of the microphone to 22,050 Hz.

**Figure 3:**
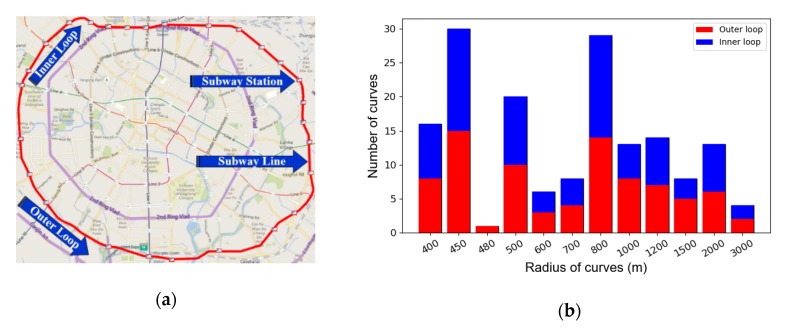
Line 7 of the Chengdu Metro, China: (**a**) Overview; (**b**) Radius of curves.

In this study, all tests were performed on Line 7 of the Chengdu Metro, China, which is a loop subway line. Its layout is shown in Figure 3a. This line covers 38.61 km and 31 stations, and it started operations in December 2017. The trains run along the outer and inner loop, with a maximum speed of 80 km/h. Because this is a loop line, it contains a large number of curve sections (166 curves). The radius distribution of these curves is presented in Figure 3b. It is challenging to maintain the track structures in good conditions due to the high number of curves, and the squeal that typically occurs along the curves is one of the most significant problems.

**Figure 4:**
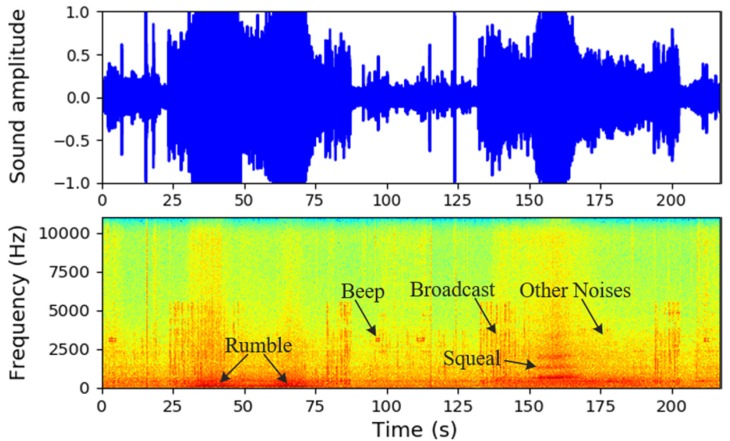
Data collection with the smartphone.

The data used in this study were collected on 2 August 2019, and 1 October 2019, before and after rail grinding. There were more abnormal events in the dataset before rail grinding. The data from August was used to train and test the multi-classification model, and to justify the need for rail grinding. The data measured on both days were compared. When training the model, we manually labeled the audio sequence into five groups, including ‘Other noises’, ‘Broadcast’, ‘Squeal’, ‘Rumble’, and ‘Beep’. Here, ‘Broadcast’ refers to the official broadcast by the subway system or passengers’ voices. ‘Squeal’ is an intense noise generated by the relative movement between wheel and rail. ‘Rumble’ refers to a low heavy sound when the train passes a specific area. ‘Beep’ is the alarm sound when a door is opened or closed. ‘Other noises’ refers to a sound which cannot be categorized into the above four classes. The time-frequency characteristics of these five classes of noise are presented in Figure 4.

## 4. Model Approach

### 4.1. Data Segmentation and Time Window

Differences in sensor sampling frequencies make it difficult to identify the corresponding relationship among the multi-source signals. In this context, data segmentation is a typical method to preprocess continuous data and capture embedded features. This approach has been frequently implemented in activity recognition, such as in speech [38] and human activity [39] recognition. Therefore, we adopted the moving time-window method to segment the signals in our study. During data segmentation, there were two crucial parameters to be determined the size of the time window and the overlap between two adjacent windows. To avoid the duplication of data interference with statistical analysis, the overlap parameter was set to 0. That is, there was no overlap between two adjacent windows. Although the window method is normally used in data segmentation, there is no clear consensus on which window size should be employed [39]. The characteristics of vehicle interior noise are different from other audio signals. Therefore, we cannot use the window sizes used in speech recognition as a reference. Generally, small windows allow for on-point activity detection with a few resources and low energy costs. In contrast, large windows are usually considered to identify complex activities. To obtain the optimal window size for vehicle interior noise multi-classification, we leveraged the Shannon entropy and the actual requirements when labeling the training data manually.

We assumed that under the optimal window size, the system carries more information than under other situations [40]. The Shannon entropy is a method commonly used to describe the average information of a system, and it can be written as:

(1) $$\;H=-\overset{m}{\underset{i=1}{\sum }}p\left(x_{i}\right)\log_{2}p\left(x_{i}\right),$$

where $x_{i}$ denotes the *i*th event; m represents the total number of events; and $p\left(x_{i}\right)$ is the probability when $x=x_{i}$ and $\overset{m}{\underset{i=1}{\sum }}p\left(x_{i}\right)=1$. To obtain the optimal window size, the vehicle interior noise signal was first divided into a series of segment sequences according to different window sizes. The standard deviation of each segment was calculated to describe the state of the segment. Consequently, standard deviation sequences corresponding to different window sizes were available. It was then assumed that all values of standard deviation fall within the range of (0, A], where A is the maximum standard deviation under different window sizes. After that, this interval was equally divided into m sub-intervals, where the *i*th sub-interval can be written as $\left(a_{i},a_{i+1}\right]$, $a_{1}=0$, and $a_{m+1}=A\;$. Thus, the optimization model for time window size can be described as:

(2) $$\max H\left(n\right)=-\overset{m}{\underset{i=1}{\sum }}p_{i}\left(n\right)\log_{2}p_{i}\left(n\right),$$

where n is the time window size, and $p_{i}\left(n\right)$ is the probability of standard deviation values to fall into the range of $\left(a_{i},a_{i+1}\right]$ when the time window size is n  . In this study, the optimal time window size was obtained from an extensive number of samples. The size of the windows ranged from 0.1 to 64 s, and the total number of samples was 200. For a higher classification accuracy, more attention should be paid to small windows. To obtain those samples, logarithm interpolation was used. For all samples, the next sample is always $10^{\left(\log_{10}64-\log_{10}0.1\right)/200}$ times the previous one. By calculating the Shannon entropy considering all 200 sizes, we obtained the maximum entropy and its corresponding window size.

### 4.2. Data Balance Using the Synthetic Minority Oversampling Technique (SMOTE)

**Figure 5:**
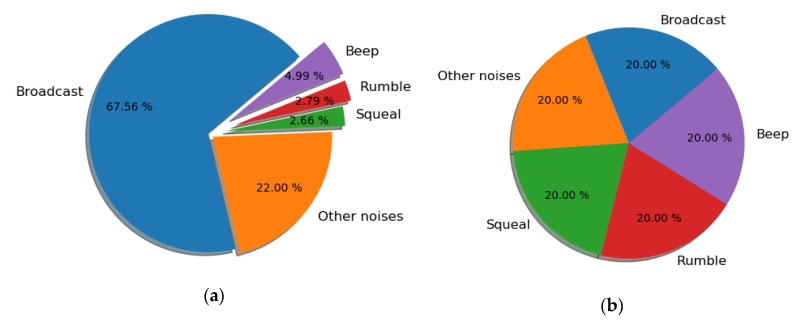
Data (**a**) before and (**b**) after synthetic minority oversampling technique (SMOTE) balance.

The pie chart in Figure 5a shows the proportion of the five categories of vehicle interior noise studied in this work. The most frequent event is ‘Broadcast’, which accounts for 67.56% of all vehicle interior noise events. ‘Other noises’ is the next most frequent event, at approximately 22%. ‘Beep’, ‘Squeal’, and ‘Rumble’ represent smaller percentages of the vehicle interior noise events, at 4.99%, 2.79%, and 2.66%, respectively. These results indicate that there is a severe class imbalance, which could significantly undermine most standard classification learning algorithms [41].

In this study, we adopted the synthetic minority oversampling technique (SMOTE) to overcome data imbalance. Generally, the class imbalance can be addressed by: (1) synthesizing new minority class instances; (2) oversampling minority class; (3) under-sampling majority class; and (4) tweaking the cost function to enhance the importance of misclassification of minority instances. The SMOTE used in this study utilizes the first solution because increasing the number of minority classes is better than merely duplicating minority classes, which has stronger robustness and generalization ability. This technique returns the original samples and an additional number of synthetic minority class samples. The SMOTE takes samples from the feature space of each minority class and its k nearest neighbors and generates new instances that combine the features of the target classes with the features of their k neighbors. Therefore, it increases the features available for each category and makes the samples more general. In this study, we increased the percentage of ‘Other noises’, Squeal’, ‘Rumbel’, and ‘Beep’ to be the same as ‘Broadcast’ via SMOTE when training the multi-classification model, as shown in Figure 5b.

### 4.3. Features

In ML, features are individual measurable properties of an observed phenomenon [42]. Selecting informative, independent, and discriminating features is a crucial process in classification or regression. The 45 features implied in this study are shown in Table 1. The feature sets include low-level signal properties (f1–f9) and Mel-frequency spectral coefficients (MFCCs) (f10–f45) [27].

Table 1 defines the features of low-level signal properties (f1–f9). N is the sample number of one segment; k refers to the *k*th sample point; x is the time-series signal; and X denotes the spectrum of Fourier transform (FT); sign( ) is the sign function; TH is the threshold, which takes the value of 0.85 in the definition of f6; P(k), which is shown in the definition of f8, is the probability distribution of the power spectrum $S\left(k\right)={\left|X\left(k\right)\right|}^{2}$. Moreover, MFCCs are features commonly used in speech and speaker recognition [38]. In this study, the first 12 MFCCs coefficients (f10–f21) were used to obtain more information from the audio segments. Because the audio signals vary intermittently, it is necessary to add features related to the change of cepstral characteristics over time [43]. Therefore, the first- and second-order derivatives of the first 12 MFCCs (f22–f33 and f34–f45) were also calculated.

### 4.4. Feature Selection Based on IG

During data analysis, hundreds of features may be generated, many of which are redundant and not relevant to the data mining task. Removing these irrelevant features may waste vast amounts of computation time and influence the prediction results. Although experts in relevant files can select the useful features, this is a challenging and time-consuming task, especially when the characteristics of the dataset are not well known. The goal of feature selection is to find a minimum set of features so that the prediction results are as close as possible to (or better than) the original feature set.

In this study, we employed the IG as an index for feature selection. IG is a feature evaluation method based on entropy and is widely employed in the field of ML [44]. In feature selection, IG is defined as the complete information provided by the features for the classification task. IG measures the importance of features as:

(3) IG(S,a)=E(S)−E(S|a),

where IG(S,a) is the IG of the original feature set S for feature a; E(S) is the entropy for the feature set without any change; and E(S|a) is the conditional entropy for the feature set, given feature a. The conditional entropy E(S|a) can be written as:

(4) $$E\left(S|a\right)=\underset{v\in a}{\sum }\frac{Sa\left(v\right)}{S}*E\left(Sa\left(v\right)\right)\;,$$

where $\frac{Sa\left(v\right)}{S}$ is the categorical probability distribution of feature a at v∈a, and E(Sa(v)) is the entropy of a sample group where a has the value v. The greater the value of IG(S,a), the more critical is a for the classification model.

### 4.5. Multi-Classification Model for Vehicle Interior Noise Based on XGBoost

XGBoost was designed based on gradient boosted decision trees [45]. We chose XGBoost due to its computation speed and model performance, which have been verified by a previous study [22]. As an ensemble model of decision trees, the definition of the XGBoost model can be written as:

(5) $${\hat{y}}_{i}=\overset{K}{\underset{k=1}{\sum }}f_{k}\left(x_{i}\right),$$

where K is the total number of decision trees, $f_{k}$ is the *k*th decision tree, and ${\hat{y}}_{i}$ is the prediction result of sample $x_{i}$. The cost function with a regularization term is given by [45]:

(6) $$L\left(f\right)=\overset{n}{\underset{i=1}{\sum }}l\left({\hat{y}}_{i},y_{i}\right)+\overset{K}{\underset{k=1}{\sum }}\Omega \left(f_{k}\right),$$

with:

(7) $$\Omega \left(f\right)=\gamma T+\frac{1}{2}\;\lambda {\left||w|\right|}^{2}\;,$$

where T is the number of leaves of the classification tree f, and w is the score of each leaf. The Lasso regulation of coefficient γ and ridge regularization of coefficient λ can work together to control the complexity of the model. By expressing the objective function as a second-order Taylor expansion, the objective function at step t can be written as [46]:

(8) $$L\left(f\right)\approx \overset{n}{\underset{i=1}{\sum }}\left[l\left({\hat{y}}_{i},y_{i}\right)+g_{i}f_{t}\left(x_{i}\right)+\frac{1}{2}h_{i}f_{t}{}^{2}\left(x_{i}\right)\right]+\Omega \left(f_{t}\right),\;$$

where $g_{i}=\partial_{\hat{y}}l\left({\hat{y}}_{i},y_{i}\right)$, and $g_{i}=\partial_{\hat{y}}{}^{2}l\left({\hat{y}}_{i},y_{i}\right)$. By removing the constant term, the approximation of the objective at step t is available:

(9) $$\hat{L}\left(f\right)=\overset{n}{\underset{i=1}{\sum }}\left[g_{i}f_{t}\left(x_{i}\right)+\frac{1}{2}h_{i}f_{t}{}^{2}\left(x_{i}\right)\right]+\Omega \left(f_{t}\right).$$

By expanding the regularization term Ω and defining $I_{j}$ as the instance set at leaf j, Equation (9) can be rewritten as [47]:

(10) $$\hat{L}\left(f\right)=\overset{T}{\underset{j=1}{\sum }}\left[\left(\underset{i\in I_{j}}{\sum }g_{i}\right)w_{j}+\frac{1}{2}\left(\underset{i\in I_{j}}{\sum }h_{i}+\lambda \right)w_{j}{}^{2}\right]+\gamma T.$$

By rewriting the objective function as a unary quadratic function of leaf score w, the optimal w and the value of the objective function are easily obtained. In XGBoost, the gain is used for splitting decision trees:

(11) $$G_{j}=\sum_{i\in I_{j}}g_{i}\;,$$

(12) $$H_{j}=\sum_{i\in I_{j}}h_{i},$$

(13) $$gain=\frac{1}{2}\left[\frac{G_{L}^{2}}{H_{L}+\lambda }+\frac{G_{R}^{2}}{H_{R}+\lambda }-\frac{{\left(G_{L}+G_{R}\right)}^{2}}{H_{L}+H_{R}+\lambda }\right]-\gamma ,\;$$

where the first and second terms are the score of the left and right child tree, respectively; the third term is the score if there is no splitting; and γ is the complexity cost when a new split is added. Despite the serial relationship between the adjacent trees, the node in a certain level can be parallel during the splitting, which enables XGBoost to have a faster train speed.

## 5. Results and Discussions

In general, the parameters of an ML model can significantly impact its performance, and XGBoost is no exception. Through extensive testing and observation, we set the critical parameters of this model as follows: maximum depth of the tree (max_depth) = 6; learning rate (eta) = 0.01; minimum sum of instance weight needed in a child (min_child_weight) = 1; subsample ratio of the training instance (subsample) = 1; fraction of features (columns) to use (colsample_bytree) = 1. The ratio between the training dataset and the test dataset was set to 0.8/0.2 in this study.

### 5.1. Optimal Time Window Size and Data Balance

**Figure 6:**
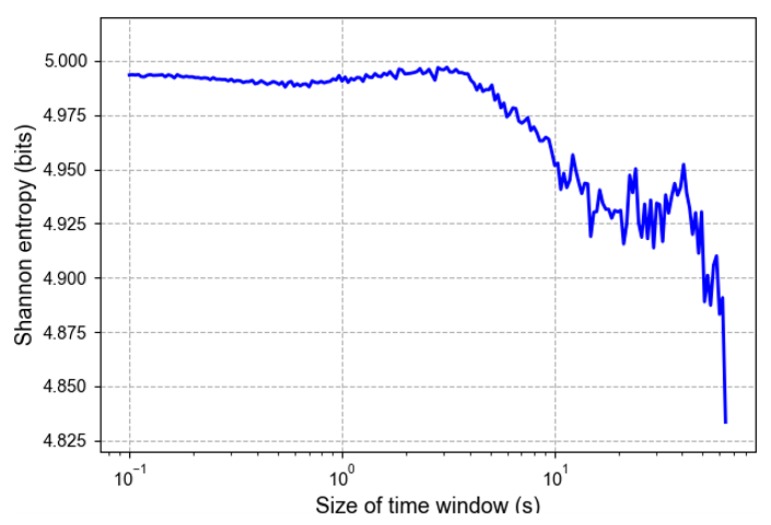
Entropy at different time window sizes.

We divided the audio signals collected from the test line into segment sequences with different time windows. Figure 6 presents the calculated Shannon entropies under different time window sizes. The Shannon entropy maintains a relatively stable state when the time window size increases from 0.1 ($10^{-1}$) to 1.58 ($10^{0.2}$) s, after which it decreases dramatically. When the time window size is 1.58 s, the Shannon entropy reached its maximum value. According to the maximum Shannon entropy hypothesis, the optimal time window size is 1.58 s. However, we maintained a relatively small window in our study to avoid a situation where one window contains different vehicle interior noise events. Therefore, we set the time window size to 1 s.

We increased the proportion of four minority classes to the same as ‘Broadcast’ with SMOTE. The performance of the multi-classification model using balanced or unbalanced training data was compared. Table 2 reports the comparison results from the perspective of precision, recall, and F1 score. ‘Support’ in this table means the total number of occurrences in each category. Data balance increased the precision of ‘Broadcast’ and decreased its recall. In contrast, it decreased the precision and increased the recall of minority classes, namely ‘Beep’, ‘Rumble’, ‘Squeal’, and ‘Other noises’. Meanwhile, F1 scores presented a slight drop after the data balance, except for the classes of ‘Beep’ and ‘Squeal’.

**Figure 7:**
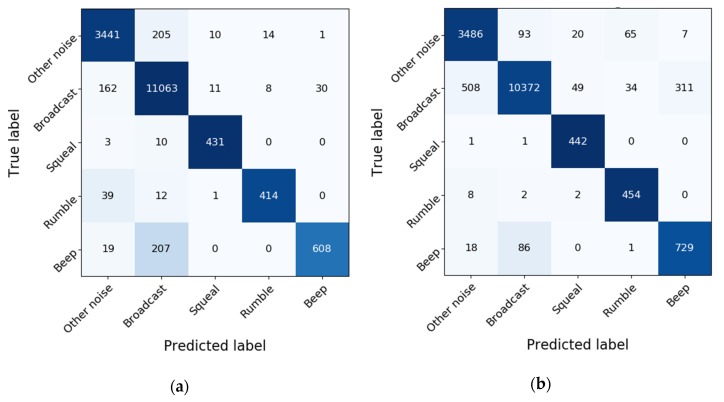
Confusion matrices of test results: (**a**) Model trained with unbalanced data; (**b**) Model trained with balanced data.

We also employed confusion matrices to describe the performance before and after the training data were balanced, as shown in Figure 7. These matrices provide insights into the errors by the classification model and distinguish the types of errors. For instance, the matrices imply that ‘Squeal’ is commonly mislabeled as ‘Broadcast’, and ‘Rumble’ is mislabeled as ‘Other noises’. One can also notice that the data balance improves the identification of the performance of minority classes such as ‘Beep’, ’Rumble’, and ‘Squeal’. ‘Squeal’ and ‘Rumble’ have a strong relationship with vehicle-track conditions, which is a major concern in our research. It is therefore desirable to detect all ‘Squeal’ and ‘Rumble’ events. Therefore, we balanced the training dataset via SMOTE to improve the recall of ‘Squeal’ and ‘Rumble’, despite the slight decrease in precision.

### 5.2. Feature Selection Based on the Importance Score

The importance was calculated explicitly for each feature by using the inbuilt feature importance property of XGBoost algorithm. The scores for features indicate how useful they were in the construction of the model and allows features to be ranked and compared with each other. Besides, a mutual information-based feature selection method is also used to verify the results of the importance-based method. In contrast to the importance score, the calculation of mutual information does not depend on the classifiers, but only considers the statistical characteristics of the input features and target variables.

**Figure 8:**
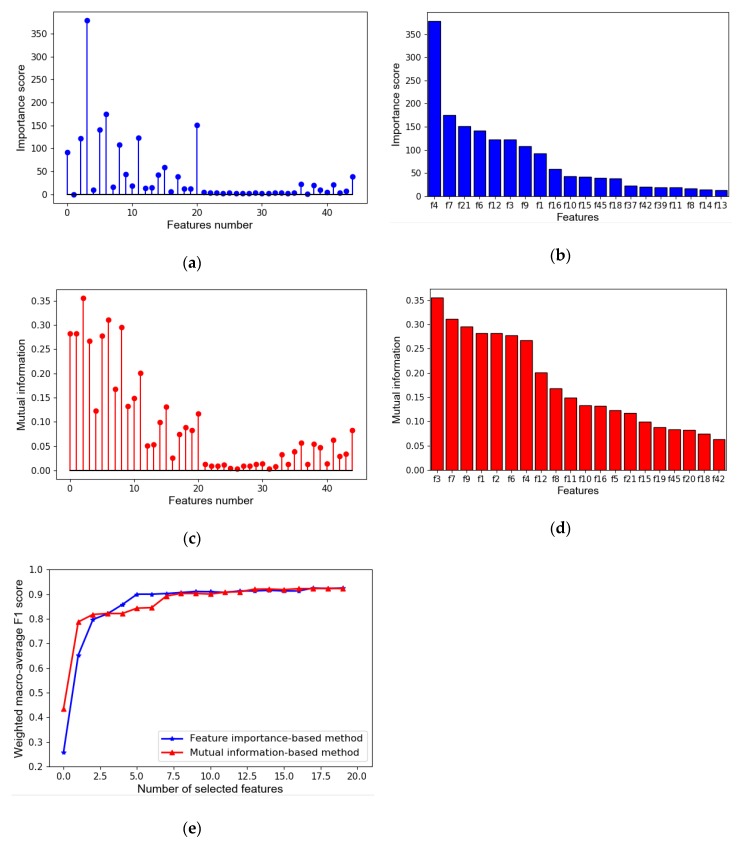
Illustration of feature selection based on different methods: (**a**) importance score of all the features; (**b**) importance score of the top 20 features; (**c**) mutual information of all the features; (**d**) mutual information of the top 20 features; (**e**) comparison of results of the two feature selection methods.

In our classification model, 45 initial features were considered. Figure 8a shows the feature importance scores calculated by gain [45]. The importance scores of different features vary greatly, ranging from 0 to 378. The spectral centroid, denoted as f4, ranks first. In contrast, the importance score of f2, root mean square (RMS) of segments, equals zero, which means that it was not used during the training process. Figure 8a also shows that the low-order features and first 12 MFCCs are essential in the classification task. The results of the feature importance analysis indicate that the contribution of different features to the model varies greatly. Thus, feature selection is necessary to improve the performance of the model and speed of calculations. Figure 8c shows the results for 45 features calculated by the mutual information-based method. The mutual information of these features has a similar trend with that of importance score. However, the importance scores of some features are very different from their mutual information value. For example, the importance score of feature f2 is 0, but its mutual information ranks fifth among all of the 45 features. The reason is that the mutual information only considering the features and target variables cannot reflect whether the features were engaged in the establishment of the classification model.

First, all 45 features were sorted in descending order of importance and mutual information, respectively. Figure 8b,d show the histograms of the top 20 features in descending order of the importance score and mutual information independently. We then constructed 20 feature sets incrementally with top 1, top 2, …, and top 20 features. Furthermore, the classification results with different features sets were compared, as shown in Figure 8e. There, the weighted macro average F1 score, $F1_{wm}$, was used to evaluate the performance of the multi-classification model, and it can be defined as follow:

(14) $$F1{\;}_{wm}=\frac{\sum_{i=1}^{N}F1_{i}\times w_{i}\;\;}{N}\;,\;$$

where N is the total number of classes, in this study N=5; $F1_{i}$ is the F1 score of the *i*th class; and $w_{i}$ is the weight of the *i*th class and there is $\overset{N}{\underset{i=1}{\sum }}w_{i}=N$. Because this study mainly focuses on ‘Squeal’ and ‘Rumble’ we set both their weights to 1.3, and the weights of ‘Other noises’, ‘Beep’, and ‘Broadcast’, to 0.8. The value of $F1{\;}_{wm}$ varies from 0 to 1. The closer the weight is to 1, the better the model performs. The red line in Figure 8e corresponds to the classification results of 20 feature sets constructed by the mutual information-based feature selection method, and the blue line corresponds to that by the feature importance-based method. The results in Figure 8e show that $F1_{wm}$ by both feature selection methods increased rapidly when the feature set expanded from the top 1 to the top 8 features. Afterward, $F1_{wm}$ remained stable. The comparison of the results of the two methods indicates that the mutual information-based method performed better than the importance-based one when the number of selected features was less than 4. However, when the feature set expanded from the top 4 to the top 11, the importance-based method performed better. Then, the continuous increase in the number of the features selected causes no obvious difference between the performances of the two methods. According to the analysis, the set with the top 10 features selected by the importance-based method was employed in this study, the $F1_{wm}$ of which reached 0.91.

### 5.3. Comparisons with Other Methods

To validate the performance and execution speed of the XGBoost-based classifier used in our study, we conducted a comparison with other commonly used classifiers, including the K-nearest neighbors, decision trees, random forest, gradient boost, extra trees, AdaBoost, and artificial neural network (ANN) classifiers. This study ran all classifiers on the same computer and with the same training and testing data set. Table 3 shows the comparison results of $F1_{wm}$ and running time. The $F1{\;}_{wm}$ value of the gradient boost ranked first at 0.925. However, training and testing the gradient boost classifier also consumed the longest running time, 340.31 s, which was approximately 22 times longer than the time needed by the XGBoost classifier. In contrast, the K-nearest Neighbors presented the fastest computing speed and one of the lowest $F1{\;}_{wm}$. Besides, the accuracy and precision of different models are provided in Table 3. The accuracy and precision share a similar trend with $F1{\;}_{wm}$. The comparison with other classifiers depicts that the XGBoost model shows a good performance in accuracy and execution speed.

### 5.4. Case Studies to Extend the Model Application Scenarios

**Figure 9:**
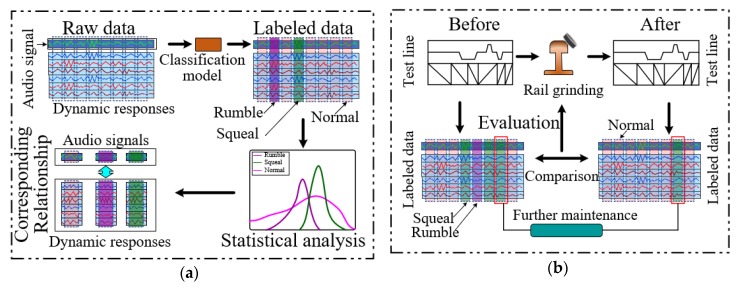
Schematics for case studies: (**a**) statistical analysis of vehicle interior noise and dynamic responses; (**b**) abnormal events detection and rail grinding effect evaluation using the XGBoost multi-classification model.

In this paper, we provided two case studies to extend the application scenarios. First, we conducted a statistical analysis to investigate the relationship between the vehicle interior noises and the dynamic responses of the car body with multi-source data collected by smartphones. After that, we used the proposed multi-classification model to detect abnormal interior noise events and evaluate the effect of rail grinding for guiding the implementation of maintenance work. Figure 9 illustrates the schematics of both case studies in this work.

In the first case study, about 10 h of onboard monitoring data collected by smartphones were used. As shown in Figure 9a, the audio signals of the vehicle interior noise were fed into the multi-classification model established in this work. According to the classification results, the raw data were labeled into three categorizations: ‘Squeal’, ‘Rumble’, and ‘Normal’. ‘Normal’ contained all other events except for ‘Squeal’ and ‘Rumble’ events. Then, statistical analyses for the dynamic responses corresponding to different vehicle interior noise were performed. This case study aimed to investigate the causes of the abnormal noise events and find out the solutions through the statistical analysis results.

**Figure 10:**
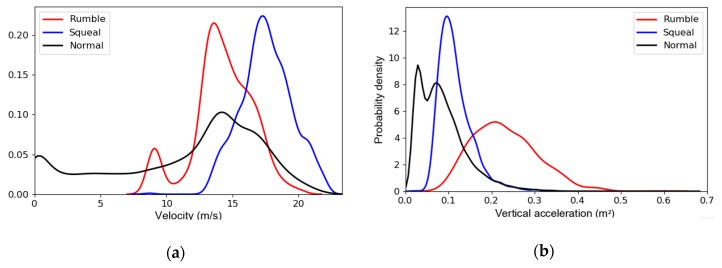
Statistical analysis of vehicle interior noise and dynamic responses: (**a**) The probability distribution curves of running speed (*v*); (**b**) The probability distribution curves of vertical acceleration ($a_{v}$).

For ‘Squeal’, ‘Rumble’, and ‘Normal’, the probability distribution curves of running speed (v) and vertical acceleration ($a_{v}$) of the car body are presented in Figure 9a,b, respectively. The vehicle speed v used here was not measured directly but obtained by the first-order integration of the longitudinal acceleration $a_{l}$ [47], which can be written as follows:

(15) $$v=\int_{0}^{t}a_{l}dt+v_{0},$$

where t denotes the time; $v_{0}$ is the initial velocity. Since the integration begins when the subway train starts, $v_{0}$ equals to 0. The probability distribution curves in Figure 10a shows that ‘Squeal’ usually occurs at higher running speed compared with ‘Normal’ and ‘Rumble’. This also suggests that we can reduce the occurrence of ‘Squeal’ by adjusting the operating speed of the train. In contrast, ‘Rumble’ occurs at a slower speed and higher vertical vibration level compared to ‘Squeal’, as shown in Figure 10b. This phenomenon implies that the occurrence of ‘Rumble’ is related to the resonance of the car body, which may be avoided by optimizing the structure of the car body.

**Figure 11:**
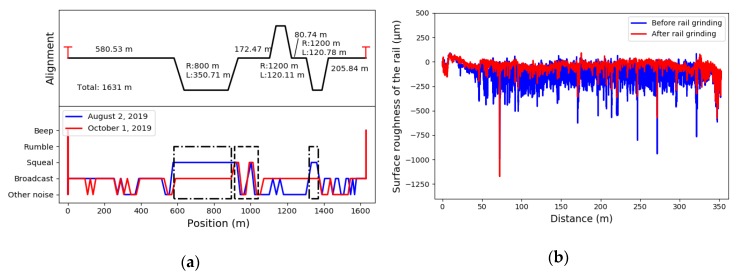
Abnormal events detection and rail grinding effect evaluation using the XGBoost multi-classification model: (**a**) track alignments of the test section and the identification results before and after rail grinding; (**b**) the surface roughness of the rail before and after rail grinding.

The schematic of the second case study is presented in Figure 9b. The test interval selected in this study was between two adjacent stations with a length of 1631 m. The track alignment of the test interval is presented in the upper plot of Figure 11a. There are three curves in the test interval, the radii of which are 1200 m, 800 m, and 800 m. This case study aimed to test the capacity of this model for identifying abnormal noise events, evaluating the effect of rail grinding, and providing information relevant to designing a future maintenance plan.

The authors first collected multi-source data with the onboard smartphone on 2 August 2019. The results of the multi-classification model are depicted in the lower plot of Figure 11a with a blue line. The results indicate that ‘Squeal’ occurred in the positions from 580 to 890 m, 910 to 1040 m, and 1320 to 1370 m. It can be seen that the figure the sections where ‘Squeal’ occurs have a high overlap ratio with the curve sections, especially the curve section with a radius of 800 m. According to the classification results and design information, we can make a preliminary conclusion that the sharp curves are the main causes of ‘Squeal’. The results also indicate the need for rail grinding or other corresponding maintenance measures.

Then, a scheduled rail grinding of the test interval was done on 21 August 2019. The surface roughness of the rail before and after rail grinding presented in Figure 11b indicates that rail grinding reduced the roughness of the rail surface effectively. Since reducing the rail roughness, that is, the unevenness on the tread of the rail benefits improving the rail-wheel contact relationship, rail grinding is a common measure for eliminating the abnormal noise and vibration of subway trains.

Another onboard test was conducted on 1 October 2019, to verify the effects of the maintenance work. The corresponding classification results after the rail grinding are displayed in red in the lower plot of Figure 11a. It can be seen that after rail grinding, the ‘Squeal’ was eliminated at 580–890 m and 1320–1370 m. However, the ‘Squeal’ at 910–1040 m remained. The results illustrate that rail grinding eliminated ‘Squeal’ at circular curves effectively. Nevertheless, it showed no apparent effect on the occurrences at transition curves and straight-line sections, which shows that there exist some other factors that lead to ‘Squeal’ in these sections. Thus, future maintenance work should focus on the section from 910 to 1040 m. This case study demonstrates the potential of applying the proposed multi-classification model in evaluating the effect of rail grinding and providing more information about the track conditions to making a further rail maintenance plan.

## 6. Conclusions

This study proposed a vehicle interior noise multi-classification model based on the XGBoost method and onboard smartphone data. By considering the Shannon entropy, a 1-second time window was selected to perform the data segmentation task. The comparison between the performances before and after the training data was balanced demonstrated that data balancing can promote the recall of minority classes but decrease the precision of their results. Feature importance analysis results show that features calculated from the spectrum of the Fourier transform and the first 12 MFCCs are the most essential among all features. By comparing and analyzing the results of importance-based and mutual information-based methods, this study selected the top 10 features in importance score to form the features set, whose $F1{\;}_{wm}$ reached 0.91. Then, the comparison between the XGBoost and other commonly used classifiers showed that the proposed XGBoost-based classification model presents a faster computing speed while maintaining a good performance. The case studies verified that the proposed multi-classification model has the potential to investigate the correlation between abnormal vehicle interior noise and dynamic responses of the train. Moreover, the capacity of the model to monitor abnormal noise events and evaluate the effect of rail grinding was also proved.

There are a few directions for future research. A more detailed classification of vehicle interior noise could be developed based on specific track-vehicle conditions so that this model would be suitable for general cases. Furthermore, more experiments are needed to explain the performance among different vehicles and track slabs. Another interesting option is to investigate the relationship between abnormal noise and wheel-rail contact conditions. Furthermore, the authors intend to set up a data collection system with high-quality sensors for more accurate and reliable data.

## Figures and Tables

**Table 1:** Features used in this study.

Category	Feature		Definition
Time-domain	f1	Segment energy	$\;f1=\overset{N-1}{\underset{k=0}{\sum }}{\left|x\left(k\right)\right|}^{2}$
	f2	Root mean square (RMS) of the segment	$\;f2=\sqrt{\frac{1}{N}\overset{N-1}{\underset{k=0}{\sum }}x{\left(k\right)}^{2}}$
	f3	Zero cross rate	$\;f3=\frac{1}{2}\overset{N-1}{\underset{k=0}{\sum }}\left|sign\left(x\left(k\right)\right)-sign\left(x\left(k-1\right)\right)\right|\;$
Frequency-domain	f4	Spectral centroid	$\;f4=\overset{N-1}{\underset{k=0}{\sum }}\left|X\left(k\right)\right|\cdot k/\overset{N-1}{\underset{k=0}{\sum }}\left|X\left(k\right)\right|$
	f5	Spectral bandwidth	$\;f5=\sqrt{\overset{N-1}{\underset{k=0}{\sum }}{\left(k-f4\right)}^{2}}$ [29]
	f6	Spectral roll-off	$\;f6=max\left\{\overset{m}{\underset{k=0}{\sum }}\left|X\left(k\right)\right|\leq TH\cdot \overset{N-1}{\underset{k=0}{\sum }}\left|X\left(k\right)\right|\right\}$
	f7	Spectral bandwidth to energy ratio	f7=f5/f1
	f8	Spectral entropy	$\;f8=-\overset{N}{\underset{n=1}{\sum }}P\left(k\right)\log_{2}P\left(k\right)$
	f9	Energy to spectral entropy ratio	f9=f10/f8
	f10–f21	First 12 MFCCs	
	f22–f33	First-order derivatives of f10–f21	
	f34–f45	Second-order derivatives of f10–f21	

**Table 2:** Classification reports of test results.

	The Model Trained with Unbalanced Data			The Model Trained with Balanced Training Data			
Classes	Precision	Recall	F1 score	Precision	Recall	F1 score	Support
Other noises	0.94	0.94	0.94	0.87	0.95	0.91	3671
Broadcast	0.96	0.98	0.97	0.98	0.92	0.95	11,274
Squeal	0.95	0.97	0.96	0.86	1.00	0.92	444
Rumble	0.95	0.89	0.92	0.82	0.97	0.89	466
Beep	0.95	0.73	0.83	0.70	0.87	0.78	834

**Table 3:** Comparisons between XGBoost and other classifiers.

Classifier	$F1{\;}_{wm}$	Accuracy	Precision	Running Time (s)
XGBoost	0.923	0.96	0.95	15.06
K-nearest Neighbours	0.704	0.84	0.72	**2.51**
Decision Trees	0.851	0.91	0.92	3.12
Random Forest	0.923	0.96	0.94	77.88
Gradient Boost	**0.925**	0.96	0.94	340.31
AdaBoost	0.651	0.77	0.64	67.70
ANN	0.880	0.93	0.94	173.22
